# Soluble LR11/SorLA represses thermogenesis in adipose tissue and correlates with BMI in humans

**DOI:** 10.1038/ncomms9951

**Published:** 2015-11-20

**Authors:** Andrew J. Whittle, Meizi Jiang, Vivian Peirce, Joana Relat, Sam Virtue, Hiroyuki Ebinuma, Isamu Fukamachi, Takashi Yamaguchi, Mao Takahashi, Takeyoshi Murano, Ichiro Tatsuno, Masahiro Takeuchi, Chiaki Nakaseko, Wenlong Jin, Zhehu Jin, Mark Campbell, Wolfgang J. Schneider, Antonio Vidal-Puig, Hideaki Bujo

**Affiliations:** 1 University of Cambridge Metabolic Research Laboratories, Level 4, Wellcome Trust-MRC Institute of Metabolic Science, Box 289, Addenbrooke's Hospital, Cambridge CB2 OQQ, UK; 2 Department of Clinical-Laboratory and Experimental-Research Medicine, Toho University, Sakura Medical Center, Sakura 285-8741, Japan; 3 Department of Genome Research and Clinical Application, Chiba University Graduate School of Medicine, Chiba 260-8670, Japan; 4 Department of Biochemistry and Molecular Biology, School of Pharmacy, University of Barcelona, and Institute of Biomedicine of the University of Barcelona (IBUB), Barcelona 08028, Spain; 5 Tsukuba Research Institute, Sekisui Medical Co. Ltd., Ryugasaki 301-0852, Japan; 6 Center for Diabetes, Endocrinology and Metabolism, Toho University, Sakura Medical Center, Sakura 285-8741, Japan; 7 Cardiovascular Center, Toho University, Sakura Medical Center, Sakura 285-8741, Japan; 8 Department of Hematology, Chiba University Hospital, Chiba 260-8677, Japan; 9 Department of Endocrinology, Affiliated Hospital of Yanbian University, Yanji 133000, China; 10 Department of Dermatology, Affiliated Hospital of Yanbian University, Yanji 133000, China; 11 Department of Medical Biochemistry, Medical University of Vienna, Max F. Perutz Laboratories, Vienna 1030, Austria; 12 Department of Cellular Genetics, Wellcome Trust Sanger Institute, Hinxton, Cambridge CB10 1SA, UK

## Abstract

Thermogenesis in brown adipose tissue (BAT) is an important component of energy expenditure in mammals. Recent studies have confirmed its presence and metabolic role in humans. Defining the physiological regulation of BAT is therefore of great importance for developing strategies to treat metabolic diseases. Here we show that the soluble form of the low-density lipoprotein receptor relative, LR11/SorLA (sLR11), suppresses thermogenesis in adipose tissue in a cell-autonomous manner. Mice lacking LR11 are protected from diet-induced obesity associated with an increased browning of white adipose tissue and hypermetabolism. Treatment of adipocytes with sLR11 inhibits thermogenesis via the bone morphogenetic protein/TGFβ signalling pathway and reduces Smad phosphorylation. In addition, sLR11 levels in humans are shown to positively correlate with body mass index and adiposity. Given the need for tight regulation of a tissue with a high capacity for energy wastage, we propose that LR11 plays an energy conserving role that is exaggerated in states of obesity.

## Abstract (web summary)

Brown adipose tissue (BAT) thermogenesis is an important determinant of organismal energy expenditure in mammals. Here, Whittle *et al*. report that the protein sLR11 is a negative regulator of BAT activity in mice, repressing thermogenesis by inhibiting BMP/Smad signalling in brown adipocytes.

## Graphical abstract

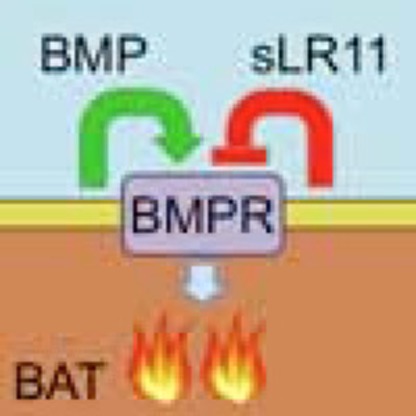

Murine studies have demonstrated that increasing heat production in brown adipose tissue (BAT) can offset obesogenic effects of a high-fat diet (HFD)^1^,^2^, whereas reducing thermogenesis exacerbates them^3^. The existence of BAT in adult humans has been ‘reconfirmed' by using modern imaging techniques and tissue biopsies^4^,^5^,^6^, increasing the importance of understanding BAT's physiological regulation. In addition, levels of detectable BAT correlate negatively with age, body mass index (BMI) and diabetes^7^, and using intermittent cold exposure to increase amounts of BAT is sufficient to increase post-prandial energy expenditure and improve insulin sensitivity^8^. Collectively, these findings support the hypothesis that thermogenesis in BAT is an important factor contributing to human metabolism. Numerous secreted and circulating factors have been identified to date with the ability to enhance the amount and/or activity of BAT^9^. Given the need for tight regulation of a tissue with such a high capacity for energy wastage, it is somewhat surprising that comparatively few negative regulators have been described. Changes in levels of LR11 in humans have primarily been associated with Alzheimer's disease, linked to its potential role in amyloid processing in the brain^10^. More recently, LR11 has been shown to possess the required properties of a functional lipoprotein receptor and the soluble form (sLR11) has been shown to facilitate increased vascular smooth muscle cell migration in response to injury, hinting at an equally important peripheral role^11^,^12^,^13^. LR11 has also been shown to regulate lipoprotein lipase (LPL), a key enzyme regulating the supply of lipid to BAT, by coordinating both its anterograde and retrograde vesicular transport^14^,^15^.

Here we show that mice lacking LR11 are protected from diet-induced obesity because of a hypermetabolic phenotype, facilitated by enhanced thermogenic gene expression in white adipose tissue (WAT) depots. Our data demonstrate that sLR11 is able to bind to bone morphogenetic protein (BMP) receptors and suppress thermogenic signalling via suppression of downstream Smad protein phosphorylation. The expression profile of *Lr11* and serum levels of sLR11 in mice increase at times of increased thermogenesis, suggesting a regulatory role of sLR11 in preventing excessive energy wastage. sLR11 levels correlate with adiposity in humans, leading us to conclude that sLR11 may be an important negative regulator of adipose tissue energy expenditure, which is dysregulated in obesity, potentially compromising metabolic responses to nutritional stimuli.

## Results

### *Lr11* ^*−/−*^ mice are hypermetabolic and protected from obesity

**Figure 1:**
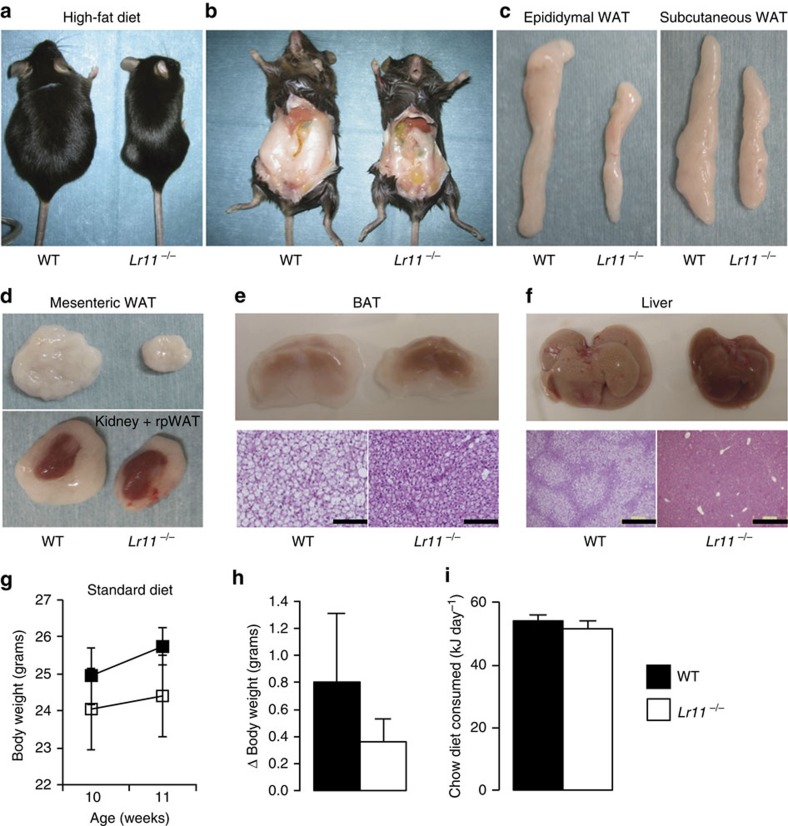
*Lr11*^*−/−*^ mice display reduced adiposity when fed a HFD. (**a**) Representative images of body size, (**b**) intra-abdominal fat, (**c**,**d**) separate white adipose depots and (**e**) brown adipose tissue and (**f**) livers with haematoxylin and eosin staining of histological sections from mice fed HFD from 8 to 16 weeks of age; scale bars, 200 μm. (**g**) Body weight, (**h**) change in body weight and (**i**) energy consumed in WT and *Lr11*^*−/−*^ mice over the course of 1 week being fed standard chow while single housed, *n*=5, data represented as means±s.e.m. (see also Table 1 and Supplementary Table 1).

**Figure 2:**
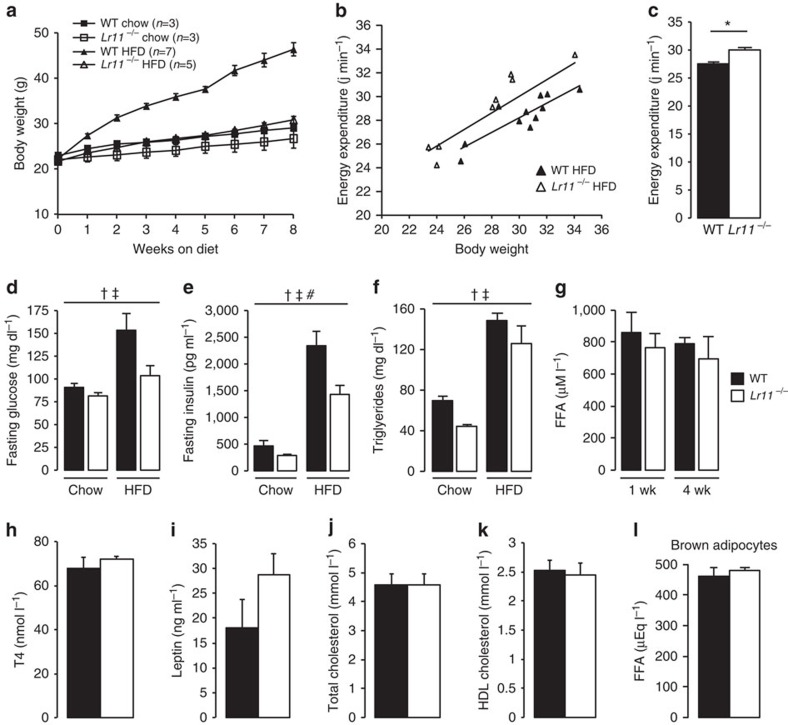
*Lr11*^*−/−*^ mice are hypermetabolic with improved serum biochemistry. (**a**) Growth curves of wild-type and *Lr11*^*−/−*^ mice fed chow and HFD from 8 to 16 weeks. (**b**) Indirect calorimetry expressed as a scatter plot showing the correlation between energy expenditure and body weight for single-housed, 12-week-old wild-type (*R*^2^=0.75) and *Lr11*^*−/−*^ (*R*^2^=0.74) mice fed HFD (60% fat) from 8 to 16 weeks; and in (**c**) also expressed as an adjusted mean energy expenditure for an adjusted mean body weight of 29.29 g, *P*-value obtained using analysis of covariance, *n*=8–11. (**d**) Fasting glucose, (**e**) fasting insulin, (**f**) fasting serum triglycerides following 8 weeks of diet administration from 8–16 weeks old. (**g**) Fasting free-fatty acids following 1 and 4 weeks of HFD from 8-weeks-old mice and (**h**–**k**) serum measurements of hormones and lipids in 16-week-old male wild-type and Lr11 ^*−/−*^ mice fed HFD from 8 to 16 weeks, *n*=5–6. (**l**) Lipoprotein lipase activity in conditioned media from mature adipocyte cultures from murine BAT of WT and Lr11^*−/−*^ mice, using free-fatty acid production as a readout (*n*=3–4). Data are presented as means±s.e.m. †, ‡ and # denote significant effect of genotype, diet and genotype*diet interaction respectively, obtained using 2-way ANOVA. (See also Supplementary Table 2). FFA, free-fatty acid.

We previously generated a mouse lacking LR11 to examine its role in vascular smooth muscle cells *in vivo* and that of the cleaved soluble form (sLR11) *in vitro*^11^,^16^. These studies highlighted a tendency for *Lr11*^*−/−*^ mice to be lighter than their wild-type (WT) littermates, and as such our central hypothesis was that LR11 was in some manner acting as a negative regulator of metabolism. To formally assess differences in body weight, WT and *Lr11*^*−/−*^ mice were fed standard chow or HFD to maximally expose differences in weight gain. The body weight of WT 8-weeks-old mice on chow diet was similar to those of *Lr11*^*−/−*^ mice; however, the size of the retroperitoneal and reproductive fat pads were significantly reduced (Table 1). In contrast, HFD-fed *Lr11*^*−/−*^ mice had markedly lower body weights than WT controls, a large portion of which was accounted for by reductions in the weights of WAT and liver (Table 1 and Supplementary Table 1). Reduced adiposity after 8 weeks of HFD was clearly evident from anatomical examinations (Fig. 1a–f) and histological analyses revealed that *Lr11*^*−/−*^ mice had smaller lipid droplets in BAT (Fig. 1e) and were protected from hepatic steatosis (Fig. 1f). Analysis of weekly energy balance on chow diet confirmed no significant difference in rate of body weight change or daily food intake in 10–11-week-old mice (Fig. 1g–i). Longer term monitoring of weight change in a separate study indicated a clear increase in weight gain in WT mice fed a HFD, with a high rate of divergence from the persistently lean *Lr11*^*−/−*^ mice observed by the comparative 10-weeks-old time-point (Fig. 2a). Averaged daily food intakes over the course of this study again confirmed no difference between genotypes (WT=59.9±1.1, *Lr11*^*−/−*^=60.5±1.2 kJ day^−1^) or HFD (WT=70.1±3.8, *Lr11*^*−/−*^=70.1±2.9 kJ day^−1^). We hypothesized that increased energy expenditure was facilitating the reduction in the rate of weight gain in *Lr11*^*−/−*^ mice fed HFD. Assessment of energy expenditure using indirect calorimetry confirmed that *Lr11*^*−/−*^ mice had a higher metabolic rate than WT mice when fed HFD (30±0.4 vs 27.5±0.3 J min^−1^) (Fig. 2b,c). *Lr11*^*−/−*^ mice also displayed improved serum biochemistry profiles, with lower fasting glucose, insulin and triglyceride levels, particularly on HFD (Fig. 2d–f). Other circulating factors such as free-fatty acids, T4, leptin and serum cholesterol were not significantly altered (Fig. 2g–k), nor were any significant alterations in LPL activity observed in Lr11^*−/−*^tissues (Fig. 2l).

### *Lr11* ^*−/−*^ mice have increased thermogenic markers in WAT

**Figure 3:**
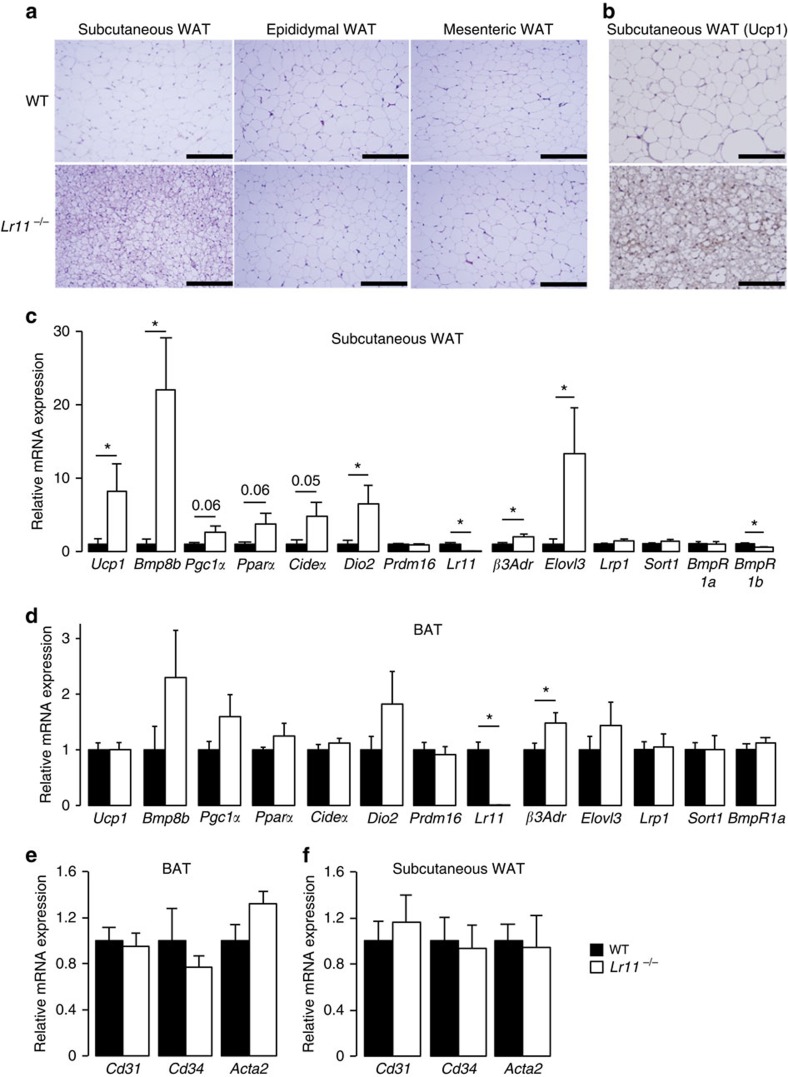
*Lr11*^*−/−*^ mice exhibit increased browning of adipose tissue. (**a**) Representative haematoxylin and eosin stained sections from WAT depots in 16-week-old wild-type and *Lr11*^*−/−*^ mice fed HFD from 8–16 weeks; scale bars, 200 μm. (**b**) UCP1 staining and (**c**) metabolic gene expression in subcutaneous WAT and (**d**) BAT and (**e**) Vascular and smooth muscle cell marker gene expression in BAT and (**f**) subcutaneous WAT of the same animals, *n*=7–8). Data are presented as means±s.e.m, *=*P*<0.05 obtained using one-way ANOVA followed by Tukey *post-hoc* test.

LR11 is known to have a predominantly neuronal expression profile^17^. However, analysis of *Lr11* mRNA expression in WT adipose tissue revealed similar levels to those found in brain, with real-time quantitative PCR ct values of 26.9±4.1 in brain versus 26.6±0.6 in BAT and 28.7±0.8 in subcutaneous WAT (scWAT) (*n*=8). We therefore examined whether phenotypic changes in adipose tissue may be driving the metabolic shift observed in *Lr11*^*−/−*^ mice. Reduced lipid content in *Lr11*^*−/−*^ BAT on HFD (Fig. 1e) suggested that the tissue was more metabolically, and thus thermogenically, active. Examination of scWAT revealed smaller, less lipid replete adipocytes (Fig. 3a), suggesting either reduced lipid storage or increased lipid oxidation. Previous studies have shown that scWAT has a high propensity for ‘browning' in response to pharmacological and nutritional signals^18^. Therefore, we performed immunostaining, which confirmed significantly higher levels of uncoupling protein 1 (UCP1) than in WT tissue (Fig. 3b). Microarray analysis of gene expression revealed significant increases in thermogenic genes, particularly in *Lr11*^*−/−*^ versus WT scWAT (Supplementary Table 2). Based on histological and microarray data we hypothesized that increased thermogenesis in adipose tissue could explain the increase in metabolic rate in *Lr11*^*−/−*^ mice. Increased expression of known key thermogenic factors was confirmed using quantitative PCR with reverse transcription, including BMP 8b (*Bmp8b*), which we have previously shown to be an important regulator of diet-induced thermogenesis (Fig. 3c)^3^. Similar gene expression changes were observed in canonical BAT, albeit to a lesser extent (Fig. 3d), likely because of the already high levels of thermogenic gene expression making detection of further modest increases more difficult. Other VPS10p or low-density lipoprotein receptor gene family receptors such as Sortilin 1 (*Sort1*) and low-density lipoprotein receptor related protein 1 (*Lrp1*) did not display any significant compensatory regulation in either scWAT or BAT (Fig. 3c,d). No changes to markers of vascular endothelial cells or the smooth muscle cell marker, alpha actin 2 (*Acta2*), were detected in either BAT or subcutaneous WAT of Lr11^*−/−*^ mice (Fig. 3e,f).

### *Lr11* ^*−/−*^ mice are more thermogenic following high-fat diet

**Figure 4:**
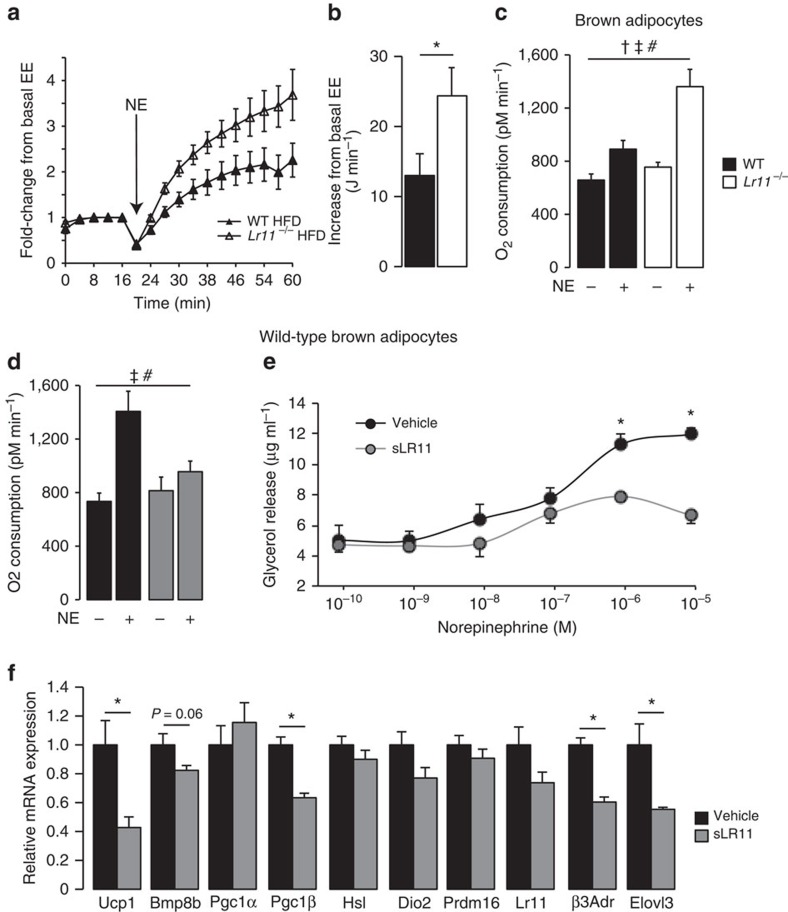
Soluble LR11 inhibits metabolism by inhibiting thermogenesis in brown adipocytes. (**a**) NE-induced increase in energy expenditure in anaesthetised mice fed 60% high-fat diet and housed at thermoneutrality for 8 weeks, expressed as fold-change from baseline and (**b**) measured increase from baseline. Arrow indicates norepinephrine injection time, *n*=6 or 7. (**c**) Cellular respiration in differentiated primary adipocytes from BAT of wild-type and *Lr11*^*−/−*^ mice in response to 100 nM NE, *n*=9–10. (**d**) Cellular respiration in primary adipocytes from wild-type BAT treated with vehicle or 10 ng ml^−1^ of soluble LR11, *n*=9 or 10. (**e**) Lipolysis measured by glycerol release from differentiated brown adipocytes in response to increasing concentrations of norepinephrine +/− 10 ng ml^−1^ of sLR11 over 2 h, *n*=4. (**f**) Thermogenic gene expression in wild-type primary brown adipocytes, differentiated in the presence of vehicle or 100 ng ml^−1^ soluble Lr11 (*n*=4 triplicate experiments). Data presented as mean±s.e.m., *=*P*<0.05 using one-way ANOVA followed by Tukey *post-hoc* test where appropriate. †, ‡ and # denote a significant effect of genotype, treatment and genotype*treatment interaction respectively, using 2-way ANOVA, for (**d**) †=effect of sLR11 treatment, ‡=effect of NE treatment and #=treatment interaction.

Increased thermogenic gene expression in *Lr11*^*−/−*^ mice fed an HFD, coupled with the reduced body weight suggested that *Lr11*^*−/−*^ mice may have increased diet-induced thermogenesis. To functionally address changes in thermogenic capacity in response to increased energy availability, we assessed the thermogenic capacity of *Lr11*^*−/−*^ mice in response to HFD. To eliminate any effect of environmental temperature, mice were housed at thermoneutrality (30 °C) while being fed HFD for 8 weeks before measurement. In this setting, any alterations to maximum thermogenic capacity are driven by diet alone and dependent on functional BAT, as demonstrated previously using mice lacking Ucp1 (ref. 19). *Lr11*^*−/−*^ mice showed a greater increase in energy expenditure from baseline in response to norepinephrine (NE) injection compared with WT littermates, corresponding to ∼12 J min^−1^ (Fig. 4a,b). This demonstrated that HFD-fed *Lr11*^*−/−*^ mice had increased BAT recruitment and thermogenic adaptation to HFD.

### sLR11 inhibits O_2_ consumption in brown adipocytes

Based on the thermoneutral HFD study data we hypothesized that a lack of LR11 allowed animals to mount a disproportionately high thermogenic response to increased sympathetic nervous system stimulation following an increase in caloric intake. Primary brown adipocytes (which normally have high endogenous thermogenic capacity) from WT and *Lr11*^*−/−*^ mice were cultured and differentiated in the presence or absence of purified sLR11 (ref. 20). Functional thermogenic capacity was assessed by NE-induced cellular respiration, as NE is the major signal from the sympathetic nervous system that stimulates thermogenesis in brown adipocytes^21^. Differentiated WT and KO adipocytes showed similar basal respiration levels but primary adipocytes from BAT of *Lr11*^*−/−*^ mice showed a greater response to NE stimulation compared with cells from WT mice (Fig. 4c). Conversely, treatment of *Lr11*^*−/−*^ cells with sLR11 almost completely ablated the enhanced effect of NE stimulation on oxygen consumption (Fig. 4d) and sLR11 significantly reduced lipolysis in WT brown adipocytes, as indicated by glycerol release in response to maximum stimulation with NE (Fig. 4e). Opposite to tissues lacking LR11, sLR11 treatment of WT primary brown adipocytes resulted in a significant reduction in expression of multiple genes essential for thermogenesis, including *Ucp1*, *Pgc1β* and β3 adrenergic receptor (*Adrβ3*) (Fig. 4f).

### sLR11 inhibits thermogenesis via the BMP/Smad pathway

**Figure 5:**
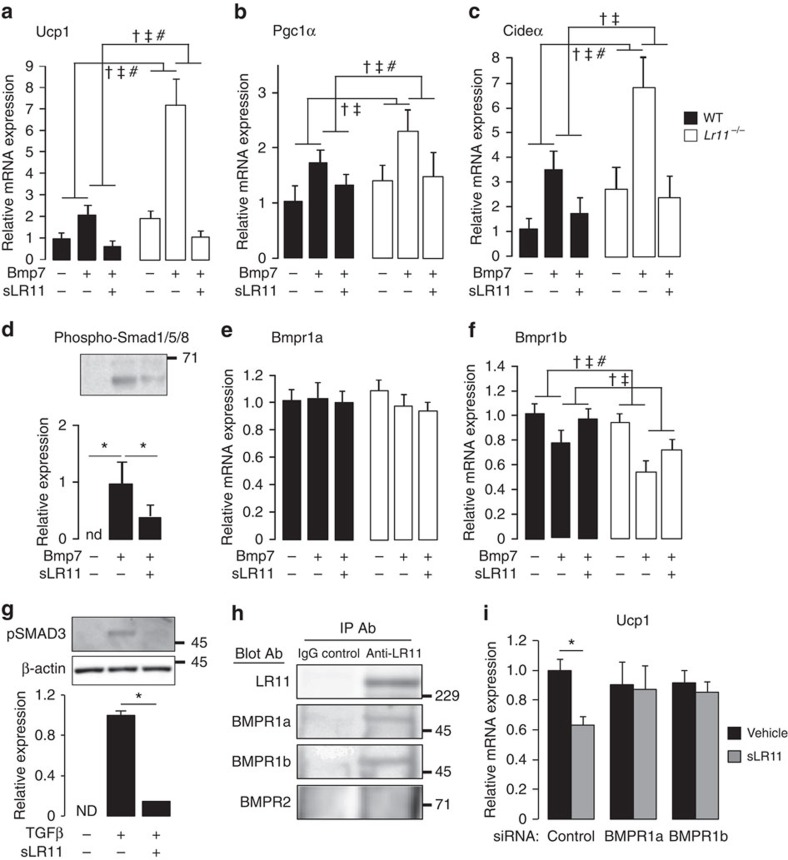
Soluble LR11 inhibits thermogenesis by inhibiting BMP signalling. (**a**–**c** and **e**,**f**) Gene expression in differentiated primary adipocytes from subcutaneous WAT of wild-type and *Lr11*^*−/−*^ mice incubated with 3.3 nM BMP7 for the first 2 days and 10 ng ml^−1^ sLR11 throughout the incubation (*n*=6). (**d**) Levels of phosphorylated Smad proteins 15 min after treatment with vehicle or 3.3 nM BMP7 in primary adipocytes subcutaneous WAT of wild-type mice, differentiated −/+ 10 ng ml^−1^ sLR11 (*n*=6). (**g**) Western blot for phospho-Smad3 in mature adipocytes cultured from subcutaneous WAT of wild-type mice treated for 30 min with 2 ng ml^−1^ TGFβ in the presence or absence of 10 ng ml^−1^ sLR11. (**h**) Western blot showing denoted proteins immunoprecipitated from membrane protein extracts from differentiated human adipocytes from scWAT with an antibody to LR11. (**i**) *Ucp1* mRNA expression following 2 h stimulation with 10 ng ml^−1^ sLR11 in mature adipocytes cultured from wild-type BAT and transfected with the indicated siRNA for BMP receptors. Data presented as mean±s.e.m., *=*P*<0.05 using one-way ANOVA followed by Tukey *post-hoc* test. †, ‡ and # denote a significant of genotype, treatment and genotype*treatment interaction respectively, using 2-way ANOVA. Blot protein weights shown in kDa. (see also Supplementary Fig. 2).

Bmp7 and its downstream signalling cascade, which requires phosphorylation of Smad proteins, has been identified as a key mechanism regulating the formation of BAT and recruitment of ‘beige' adipocytes within WAT^22^,^23^,^24^,^25^. To examine whether LR11 was interacting with this mechanism to regulate the observed browning of scWAT, we differentiated primary adipocytes from scWAT of WT and *Lr11*^*−/−*^ mice in the presence of recombinant BMP7. BMP7 is a known driver of thermogenic development and, as a member of the TGFβ superfamily, signals through BMP receptors (BMPR) to increase phosphorylation of intracellular Smad proteins^25^. Cells lacking LR11 displayed a significantly greater increase in thermogenic gene expression in response to BMP7, and this response was completely blocked in cells of both genotypes by treatment with sLR11 (Fig. 5a–c). Phosphorylation of Smads 1, 5 and 8 following BMP7 treatment of primary adipocytes was also greatly reduced by sLR11 co-treatment (Fig. 5d). sLR11 treatment was also blocked downregulation of the BMP receptor *Bmpr1b*, observed after BMP7 treatment (Fig. 5e,f) and significantly reduced phosphorylation of SMAD3 in adipocytes following stimulation with TGFβ (Fig. 5g). Further evidence for the ability of sLR11 to directly inhibit the BMP signalling pathway was provided by an immunoprecipitation assay, which showed that sLR11 was able to bind BMPR1a and BMPR1b, but not BMPR2 (Fig. 5h). Finally, reduced expression of either BMPR1a or BMPR1b in cultured brown adipocytes was sufficient to negate the repressive action of sLR11 on *Ucp1* expression (Fig. 5i).

**Figure 6:**
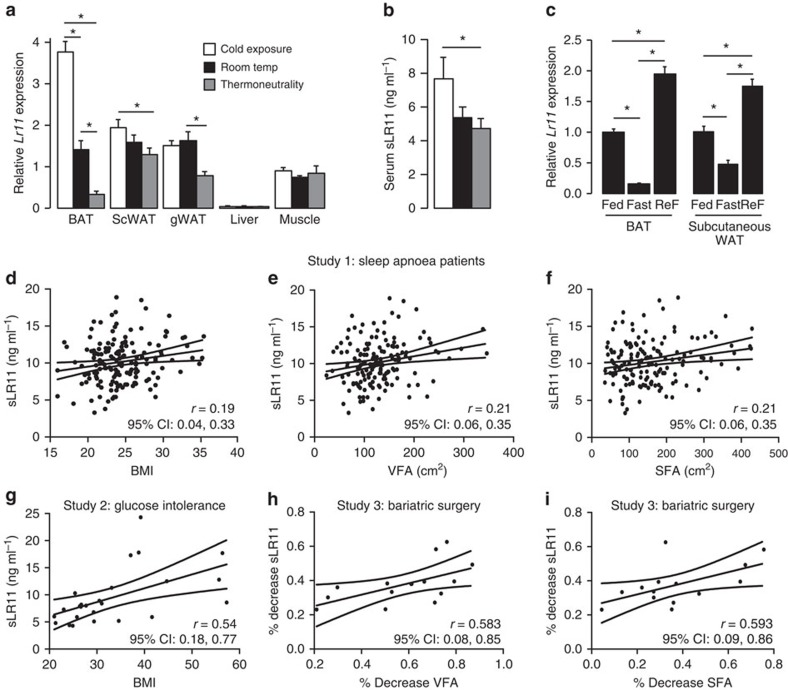
sLR11 levels are metabolically regulated and correlate with BMI and adiposity in humans. (**a**) *Lr11* mRNA expression levels in tissues from 12-week-old mice exposed to cold (5 °C), room temperature (23 °C) or thermoneutrality (30 °C) for 3 weeks (*n*=8) and (**b**) serum sLR11 levels in mice housed under the same conditions (*n*=4). (**c**) *Lr11* mRNA expression in BAT and ScWAT from fed, 24 hr fasted and 24 h re-fed mice (*n*=8), data presented as mean±s.e.m., *=*P*<0.05 using one-way ANOVA followed by Tukey *post-hoc* test. (**d**) Serum sLR11 levels in patients with sleep apnoea correlated with BMI, (**e**) VFA and (**f**) SFA (*n*=156). (**g**) Serum sLR11 levels in patients with type 2 diabetes mellitus or glucose intolerance-associated metabolic disorders correlated with BMI, (*n*=25). (**h**) % reduction in serum sLR11 levels in 14 obese patients 12 months after bariatric surgery correlated with % reduction to VFA and (**i**) SFA. Correlations evaluated using Pearson correlation (see also Tables 2 and 3). scWAT, subcutaneous white adipose tissue; gWAT, gonadal adipose tissue.

The cytoplasmic domain of LR11 has also been shown to function as a transcription factor^26^, leading us to test whether LR11 could regulate the transcriptional activity of known thermogenic response elements. Overexpression of LR11 resulted in a small but significant reduction in transcription driven by the *Bmp8b* promoter, a thyroid response element and the *Ucp1* promoter enhancer, but not by the *Ucp1* core promoter (Supplementary Fig. 1). We next examined the physiological regulation of LR11 and found its gene expression to be regulated in line with thermogenic activation, decreasing in both BAT and scWAT as environmental temperature rose from cold to thermoneutrality (Fig. 6a). Serum sLR11 levels followed the same pattern, reaching their highest point at 5 °C when thermogenesis is maximally activated in mice (Fig. 6b). *Lr11* mRNA was also regulated by changes to energy availability in a pattern identical to thermogenesis, increasing in the fed state and declining after fasting (Fig. 6c).

### sLR11 levels correlate with BMI and adiposity in humans

Finally, to test the relevance of our findings to human metabolism and as LR11 is highly expressed in adipose tissue, we examined the relationship between adiposity and sLR11 in two cohorts of patients. In 156 subjects with sleep apnoea (Supplementary Table 4), circulating sLR11 levels were positively correlated with age, BMI, visceral (VFA) and subcutaneous fat areas (SFA), and HbA1c and negatively correlated with smoking and high-density lipoprotein (HDL) cholesterol (Fig. 6d–f and Table 2). Multiple regression analysis of these variables showed that sLR11 levels were not significantly associated with any of them but tended to associate most with VFA, independently of other factors (Table 2). A second study of 25 patients with type 2 diabetes mellitus or glucose intolerance-associated metabolic disorders (Supplementary Table 5) showed a similar significant correlation between BMI and sLR11 levels (Fig. 6g). Importantly none of the other measured characteristics of these subjects, such as age, HbA1c or cholesterol levels, showed any correlation with sLR11 (Supplementary Table 5). This further suggested that overall body weight, and thus adiposity, was the major determinant of sLR11 levels in humans. In a third study of 14 obese patients who underwent bariatric surgery, post-operative sLR11 levels were significantly reduced after 12 months, concomitantly with reductions to BMI, VFA and SFA (Table 3). The percentage reduction in sLR11 was positively correlated with that of VFA and SFA (Fig. 6h,i). Multiple regression analysis of the two significant correlates, VFA and SFA, showed no significance between their respective contributions to the relationship (Table 3), suggesting that overall fat mass, regardless of its distribution, was driving the correlation.

## Discussion

Our data demonstrate a role for sLR11 as a negative regulator of thermogenesis, acting through the BMP/Smad pathway in adipose tissue to suppress activation of thermogenic machinery and inhibit transcription of key thermogenic genes. LR11 is regulated in tissues with inherent/adaptive thermogenic capacity, in response to changes in environmental temperature and diet, but not in other metabolic tissues such as liver (Fig. 6a). In absence of any endogenous LR11, nutritional stimuli that induce thermogenesis in scWAT are left unchecked, leading to increased recruitment of brown adipocytes, reduced lipid accumulation, hypermetabolism and overall leanness.

Our *in vitro* data support a cell-autonomous role of sLR11 in regulating adipose tissue responses to increased energy availability. This is suggested by the peptide's ability to suppress the known thermogenic signalling pathways and the lack of any significant change to energy balance on a standard chow diet. Neurological roles for LR11 have been previously described, although we hypothesize that if LR11 is required to maintain a central circuit regulating metabolic rate, it would be expected that its ablation would yield a consistent phenotype across environmental conditions. Instead metabolic rate changes only become apparent in *Lr11*^*−/−*^ mice fed HFD, when sympathetic tone to BAT and WAT depots increases and adaptive changes occur to strike a new balance between lipid oxidation and storage. In the absence of LR11 and thus sLR11, increased energy availability results in increased DIT in adipose tissue but without the appropriate anabolic response. Where WT mice are able to store additional calories as triglyceride, *Lr11*^*−/−*^ mice can only increase the metabolic arm of the response and burn them. However, even on a chow diet there is a slight tendency for *Lr11*^*−/−*^ mice to be lighter and consume slightly less food (Fig. 1g–i and Fig. 2a) and it is also highly likely that DIT is itself regulated by discrete neuronal pathways that mediate alterations to sympathetic activation of thermogenic tissues. Therefore we cannot rule out based on our current data, that LR11 may also play a central role in the initiation and maintenance of metabolic responses and energy balance.

LR11 has also been previously shown to increase vascular smooth muscle cell migration in response to injury^11^. We did not observe any alteration to vasculogenesis or angiogenesis in *Lr11*^*−/−*^mice, suggesting that perturbations in these processes are not causative of the hypermetabolic phenotype. Similarly, the documented ability of LR11 to regulate LPL activity^15^ was not evident as a mechanism by which its deletion led to hypermetabolism in our model (Fig. 2l). Our data also suggest that sLR11 levels are dependent on adipose mass in humans, increasing in line with fat mass. Clinical interventions such as bariatric surgery that lead to reductions in adiposity, also lower sLR11 levels (Table 3 and Fig. 6h,i), potentially contributing to the observed increase in metabolic rate in such patients^27^,^28^. The fact that the strongest correlation in all human subjects is between sLR11 and visceral and SFA, rather than BMI is further evidence that adipose tissue is the main site of sLR11 production and secretion.

We identify LR11 as a key metabolic regulator, which functions to maintain the adequate balance between lipid storage and oxidation in response to changing environmental conditions and particularly when thermogenic activity is increased in capable tissues. This mechanism might be exploited for anti-obesity therapies or conversely, for the treatment of conditions such as anorexia nervosa or cachexia. The apparent anabolic role of sLR11 may also be relevant for understanding its prior implication in cardiovascular disease and Alzheimer's disease^29^,^30^. While an increasing number of factors and pharmacological molecules exist that either increase the number of brown adipocytes or their thermogenic capacity^9^,^31^, only a small number of negative regulators are apparent, namely nuclear receptor-interacting protein 140 (Rip140) and the diurnally regulated Rev-erbα (refs 32, 33). It seems likely that other negative regulators must exist to ensure effective control of this energy-expensive mechanism and to ‘fine tune' the thermogenic response. Before our study, a lipoprotein receptor-mediated mechanism for regulation of BMP signalling in the regulation of thermogenesis has not been described.

## Methods

### Animals and diets

Unless otherwise stated, all data are from work on males (8–16 weeks of age). C57Bl6/J mice were purchased from Charles River. *Lr11*^*−/−*^ mice were generated as previously described^11^ on a C57Bl6/J background and compared to WT littermates. Unless stated, mice were housed in a temperature-controlled room (22 °C) with a 12-h light/dark cycle with free access to diet and water. Standard chow or HFD was administered *ad libitum* from weaning until indicated. High-fat diet consisted of 60% calories from fat (D12492, Research Diets, NJ, USA) unless otherwise stated. Thermoneutrality involved housing mice at 30 °C in a normal home cage environment. All animal procedures were approved by either the UK Home Office or the Special Committee on Animal Welfare, School of Medicine, at the Inohana Campus of Chiba University.

### Calculation of energy expenditure

Animals were placed in a monitoring system based on their home cages (Creative Scientific, Canterbury, UK) with the ability to measure oxygen and carbon dioxide concentrations using a system (Minimox) designed by Peter Murgatroyd (University of Cambridge)^34^. Oxygen consumption and carbon dioxide production was measured, with samples taken at 18 min intervals over a 48-h period. Energy expenditure was then calculated using indirect calorimetry with the Elia and Livesey constant^35^. Energy expenditure is expressed as J min^−1^ per mouse using an adjusted mean body weight. This was obtained using analysis of covariance with weight as the covariate, a robust method for comparison of groups with divergent body weight and composition^36^. High-fat diet consisting of 45% calories from fat (D12451, Research Diets, NJ, USA) was used for the calorimetric study as the consistency of the 60% fat diet is not compatible with the food hoppers in the measurement system.

### Serum biochemistry

Murine serum triglycerides and glucose concentrations were measured using Triglyceride E-test kit and glucose CII kit, respectively (Wako chemicals, Osaka Japan), according to the manufacturer's instructions. Serum insulin levels were measured using ultrasensitive ELISA kit (Morinaga, Yokohama, Japan) according to the manufacturer's instruction.

### Microarray

The four RNA sources, WT scWAT, *Lr11*^*−/−*^ scWAT, WT BAT and *Lr11*^*−/−*^ BAT, were prepared from subcutaneous WAT and BAT from WT mice and *Lr11*^*−/−*^ mice, respectively (*n*=3 each). Microarray analyses were performed by Takara Bio (Japan). Briefly, cyanine-3-labelled cRNA was prepared from 0.1 μg total RNA using the Low Input Quick Amp Labeling kit (Agilent) according to the manufacturer's instructions, followed by RNAeasy column purification (QIAGEN, Valencia, CA). Dye incorporation and cRNA yield were checked with the NanoDrop ND-2000 Spectrophotometer. An amount of 0.6 μg of cyanine-3-labelled cRNA was then fragmented at 60 °C for 30 min in a reaction volume of 25 μl containing 1 × Agilent fragmentation buffer and 2 × Agilent blocking agent following the manufacturer's instructions. On completion of the fragmentation reaction, 25 μl of 2 × Agilent hybridization buffer was added to the fragmentation mixture and hybridized to Agilent Mouse GE 8x60K Microarray for 17 h at 65 °C in a rotating Agilent hybridization oven. After hybridization, microarrays were washed 1 min at room temperature with GE Wash Buffer 1 (Agilent) and 1 min at 37 °C GE Wash buffer 2 (Agilent), then dried immediately by brief centrifugation. Slides were scanned immediately after washing on the Agilent DNA Microarray Scanner (G2565CA) using one colour scan setting for 8 × 60 k array slides (scan area 61 × 21.6 mm, scan resolution 3 μm, dye channel is set to Green PMT to 100%). The scanned images were analysed with Feature Extraction Software 10.10 (Agilent) using default parameters to obtain background-subtracted and spatially detrended processed signal intensities. The gene-tree analysis revealed six clusters based on the expression pattern of the four RNA sources. The gene expression pattern with Cluster 6: WT scWAT<*Lr11*^*−/−*^ scWAT<or=WT BAT=or<*Lr11*^*−/−*^ BAT was dominant compared with other patterns. The top 100 genes of *Lr11*^*−/−*^ scWAT>WT scWAT in cluster 6 were listed and summarized (Supplementary Table 2).

### Quantitative PCR with reverse transcription

Total RNA was isolated from cells or using Buffer RLT and purified by RNeasy Mini columns (Qiagen). RNA was isolated from ground tissues using STAT-60 reagent (TEL-TEST) followed by chloroform extraction and isopropanol precipitation. Complimentary DNA was generated from 500 ng of RNA using M-MLV reverse transcriptase and master mix (Promega) in a 20 μl reaction mixture with 2.5 mM MgCl_2_, 1.25 mM dNTPs and 5 μg ml^−1^ random hexamers at 37 °C for 1 h. Complementary DNA was diluted 75-fold and 5 μl of diluted complementary DNA was used in a 12 μl real-time PCR reaction mixture using TaqMan primers and probes or SYBR green reagent (Applied Biosystems) according to manufacturer's instructions. Reactions were run in duplicate for each sample and quantified in the ABI Prism 7900 sequence detection system (Applied Biosystems). Data are expressed as arbitrary units where expression of target genes are corrected using the geometric average of four housekeeping genes: 18S, β2-microglobulin, β-actin and 36B4 using Bestkeeper (freeware). Sequences of primers and probes used are listed in Supplementary Table 3.

### Tissue collection and histology

All animal tissues for protein or RNA extraction were snap frozen in liquid nitrogen at the time of collection unless otherwise stated and later ground to a fine powder using a sterile pestle and mortar on liquid nitrogen. Samples for histology were placed in 10% buffered formalin overnight before transfer to 70% ethanol and later embedding in paraffin. Multiple sections were stained with haematoxylin and eosin for morphological analysis.

### Immunohistochemistry

Paraffin-embedded sections (5 μm) were used for immunohistostaining. Deparaffinized sections were pretreated with 0.3% H_2_O_2_ to inactivate endogenous peroxidase. Slides were stained in the presence of 3% bovine serum albumin (BSA) with antibody against UCP1 (1:50 dilution) at 23 °C for1 h followed by horseradish peroxidase-conjugated anti-rabbit IgG secondary antibodies (Molecular Probes) at 1:200 dilution. The slides were counterstained with haematoxylin. Controls with non-immune rabbit IgG were conducted in parallel with each immunoassay procedure.

### Western blot

Powdered tissue or collected cultured cells were resuspended in lysis buffer (20 mM Tris-HCL, 150 mM NaCl, 1 mM EGTA, 1 mM EDTA, 1% Triton X-100, pH7.5) with added protease and phosphatase inhibitor cocktails according to manufacturers instruction (Sigma). After lysis, lysates were cleared by centrifugation at 10 000*g* for 10 min at 4 °C. Protein concentrations of the supernatants were determined by D_c_ Protein assay (Bio-Rad). Proteins were diluted in Laemmli buffer with 1% 2-mercaptoethanol. Proteins (20 μg) were separated by SDS–polyacrylamide gel (10%) electrophoresis and transferred to Immobilon-P membrane using a semi-dry transfer apparatus (Biorad). Membranes were blocked for 1 h at room temperature and incubated overnight at 4 °C with the indicated antibody. Bound primary antibodies were detected using peroxidase-coupled secondary antibodies and enhanced chemiluminescence (Amersham). Relative quantification of band intensities was calculated by digitally photographing exposed films and using Genesnap and Genetools software (Syngene). Uncropped scans of blots are shown in Supplementary Fig. 2.

### SMAD assays

Cultured fully differentiated adipocytes derived from the subcutaneous fat tissues of wild-type or Lr11^−/−^ mice in the presence of BMP7 for the first 2 days were prepared. After serum depletion for 16 h, the cells were incubated with or without 3.3 nM BMP7 or 2 ng ml^−1^ TGF-b in the presence or absence of 10 ng ml^−1^ sLR11 for 30 min, and then collected for the following western blot analyses. Cells were washed three times with phosphate-buffered saline (PBS) and harvested in RIPA buffer containing 0.01 mM APMSF. For immunoblotting, equal amounts of solubilized protein was subjected to 10% SDS−polyacrylamide gel after heating to 95 °C for 5 min under reducing conditions and transferred to a nitrocellulose membrane.

### Immunoprecipitation

An amount of 100 μg of membrane protein was mixed with 1 μg of anti-human LR11 antibody M3 or control IgG, and rotated for 1 h at 4 °C. The LR11/BMPRs/antibody complex was precipitated by protein A Sepharose. The proteins were released into 25 μl SDS sample buffer by heating to 95 °C for 10 min, and applied for western blot analysis using anti-BMPR1A, BMPR1B or BMPR2 antibody as described.

### Antibodies and recombinant and purified proteins

Monoclonal antibodies against phospho-SMAD1/5/8 (#9511, Lot#14, 1:1,000), SMAD3 (#C25A9, Lot#13, 1:1,000) and BMPR2 (#6979, Lot#1, 1:1,000) were obtained from Cell Signaling. Monoclonal antibodies against BMPR1A (MAB2406, Lot#UEJ0112071, 1 μg ml^−1^) and BMPR1B (MAB505, Lot#DGD0410091, 1 μg ml^−1^) were obtained from R&D Systems. Monoclonal Antibody to β-Actin was obtained from Sigma Aldrich (#A2228, Lot#052M4816V, 1:1,000). Rabbit polyclonal antibody against UCP1 was obtained from Abcam (ab23841, Lot#GR34748-1, 5 μg ml^−1^). Recombinant human BMP7 (354-BP, Lot#EOS1914031, 3 nM l^−1^) was obtained from R&D Systems. Soluble form of LR11 protein (sLR11) was purified from the medium incubated with Colo201 (B226, JCRB Cell Bank, Osaka, Japan) by affinity column using monoclonal antibody against sLR11, followed gel filtration as previously described^20^. Purified sLR11 was concentrated and used for experiments at a concentration of 10 ng ml^−1^, estimated from its biological activity in stimulating the migration of smooth muscle cells as previously described^11^.

### Culture of adipocytes

Mouse primary adipocytes were obtained from either BAT or subcutaneous WAT by excision of the fat pad followed by mincing with sterile surgical scissors and digestion at 37 °C with shaking for ∼35 min (10 ml Hanks buffered salt solution, 2% BSA, 2 mg ml^−1^ Type 2 collagenase (C6885, Sigma). Digests were filtered through a 100 μM filter and left to sit on ice for 10 min. The upper fat layer was removed and then the upper 2/3 of the supernatant washed twice in PBS before being plated. Cells were maintained and differentiated in Dulbecco's modified Eagle's medium with 10% fetal bovine serum, 20 mM L-glutamine, 100 units ml^−1^ penicillin and 100 μg ml^−1^ streptomycin with 150 μM sodium ascorbate and 4 nM insulin in the presence of 1 nM 3, 3′, 5-Triiodo-L-thyronine at 37 °C in 5% CO_2_. After 8 days, cells were fully differentiated (validated by gene expression profiles and morphological appearance), and if BMP7 and/or sLR11 were added, 3.3 nM BMP7 was present in the culture media for the first 2 days and/or 10 ng ml^−1^ sLR11 was present throughout the culture period. Cultures were routinely tested for the presence of mycoplasma contamination and decontamination/rederivation performed when necessary. Human primary preadipocytes were prepared from the stromal vascular fraction of fat tissues and cultured as described^37^. The procedures were approved by the Ethical Committee on human study, Toho University Sakura Medical Center.

### Transfection of cultured brown adipocytes with siRNA

Cultured brown adipocytes were transfected with siRNA (25 nM) specific for BMPR1A (L-040598-00-0005, Dharmacon, GE healthcare) or BMPR1B (L-051071-00-0005, Dharmacon), or with control siRNA (D-001810-10-05, Dharmacon) for 3 days according to manufacturer's instruction protocol ( https://dharmacon.gelifesciences.com/uploadedFiles/Resources/basic-dharmafect-protocol.pdf) for the siRNA transfection reagent (T-2001-01, Dharmacon), and then incubated with differentiation media for mature brown adipocytes for 2 days. After serum depletion for 16 h, the cells were incubated with or without sLR11 at 10 ng ml^−1^ for 2 h, and used for real-time PCR analyses.

*Cell lines*. Hek 293 cells were obtained from Sigma (product number 85120602). While cells were not independently authenticated, this was not a major concern as they were used only for the luciferase reporter assays and have been routinely used in our laboratory for multiple previous similar experiments without problems. Grossly the cells behaved as expected in terms of their growth and morphology and their viability and transfection efficiency were found to be excellent. As for primary cultures, cultures were routinely screened for mycoplasma contamination.

*LPL activity assay*. This assay was performed as previously described^38^. Briefly, the culture medium for adipocytes cultured for 16 h without fetal bovine serum were collected and the LPL activity was measured as below using triolein (Sigma, St Louis, MO) as the substrate. The substrate solution was prepared as follows: 100 mg of triolein and 7.5 ml of 0.2% Triton X-100 at a final volume of 7.5 ml of 1 mole l^−1^ Tris-HCl (pH 8.0) were sonicated on ice for 10 min. The LPL activity assay reaction mixture contained 50 μl of substrate solution, 25 μl of 20% fatty acid-free bovine serum albumin (pH 8.0; Sigma), 5 μl of HDL (3 mg protein ml^−1^) as a source of apo C-II and 100 μl of sample. After incubation for 2 h at 37 °C, the enzyme reaction was terminated by the addition of 10 μl of di-isopropylfluorophosphate. The amount of free-fatty acids released in the mixture was measured at 4 °C using an enzymatic method (Nescauto NEFA V2; Alfresa Pharma, Osaka, Japan).

### Cellular respiration

Differentiated primary adipocytes were placed in a XF24 bioanalyser (Seahorse Bioscience, MA, USA) in the manufacturer's designated culture/assay plate (each plate was seeded with an equal number of cells from a pool of primary adipocytes from two animals). Basal cellular respiration was measured over a period of 15 min using the oxygen consumption rate (OCR) measurement assay template. An amount of 100 nM NE was then added to the assay medium (XF assay medium, Seahorse) and respiration measured for a further 15 min using OCR measurement template. Basal and stimulated oxygen consumptions reported are the mean of three separate OCR measurements per well.

### NE-induced thermogenesis

Two individually housed mice (litter mates) were monitored simultaneously at an airflow of 1 l min^−1^. HFD-fed mice housed at thermoneutrality for 8 weeks before assessment were anaesthetized with pentobarbital (45 mg kg^−1^ i.p.). The oxygen consumption and carbon dioxide production of the mice was measured at 30 °C to obtain basal values during 15 min. Thereafter the mice were removed from the metabolic chambers for 6 min and injected subcutaneously with NE (1 mg kg^−1^) before being returned to the metabolic chambers for 45 min. Energy expenditure was calculated as described above but presented as the fold increase or mean increase, thus not requiring covariate analysis.

### Clinical study

Study 1 subjects consisted of 156 consecutive patients with sleep apnoea syndrome and study 2 consisted of 25 consecutive patients with type 2 diabetes mellitus or glucose intolerance-associated metabolic disorders at Toho University Sakura Medical Center or Yanbian University Hospital. Blood samples were collected in the morning after an overnight fast, and the biochemical data were measured using standard hospital laboratory techniques. The potential risk factors for diabetes and atherosclerosis were analysed, including age, sex, BMI, VFA and SFA, blood lipids and HbA1c for both studies; smoking, medication of diabetes, medication of hypertension, medication of dyslipidemia, history of coronary artery disease and history of cerebrovascular diseases for Study 1; fasting plasma glucose and systolic and diastolic blood pressures for Study 2. In Study 3, 14 obese subjects received bariatric surgery at Toho University Sakura Medical Center, and their VFA and SFA were measured before and 12 months after the surgery. The body weights of all patients decreased 1 year after surgery compared with those before surgery. Visceral fat areas and SFA were measured at umbilical levels of patients using computed tomography. Hypertension was defined as systolic pressure of more than 140 mm Hg or diastolic pressure of more than 90 mm Hg. Diabetes mellitus was defined as a fasting blood glucose level of more than 126 mg dl^−1^, glycosylated haemoglobin (HbA1c) of more than 6.2%, or both. Dyslipidemia was defined as serum total cholesterol of more than 220 mg dl^−1^ and triglycerides of more than 150 mg dl^−1^ in the fasting state, or both, and HDL cholesterol <40 mg dl^−1^, or a combination thereof. The patients suffering from possible malignant diseases, Alzheimer's disease, renal diseases or hepatic diseases were excluded in the study subjects. The procedures of the above human studies were approved by the Human Investigation Review Committee on human study, Toho University Sakura Medical Center or Yanbian University Hospital. Informed consent was obtained from all subjects.

### sLR11 measurement

Serum sLR11 concentrations were determined using a sandwich ELISA system. 12.5 μl of human and 2.5 μl of murine serum were used for the measurement of sLR11 by respective ELISA. Human serum sLR11 concentrations were determined using a revised procedure based on sandwich ELISA system previously reported^39^. In brief, 12.5 μl of human serum were diluted eight-fold with 1% BSA in PBS containing 0.1% Tween 20 (PBST), reacted with the capture anti-LR11mouse monoclonal antibody M3 F(ab′)2 for 2 h, then incubated with a biotinylated capture anti-LR11 rat monoclonal antibody R1 for 1 h. A murine serum soluble LR11 ELISA was performed using two mouse monoclonal antibodies, Mab93222 and Mab93213 that were established by immunization of *Lr11*^*−/−*^ mice using synthetic peptides against different epitopes of VPS10p domain of LR11 protein^40^. Briefly, 2.5 μl of murine serum were diluted 20-fold with 1% BSA in PBST, reacted with the capture Mab93222 for 2 h, and then incubated with the biotinylated capture Mab93213 for 1 h. The LR11-antibody complex was quantified with horseradish peroxidase-conjugated streptavidin using purified human and rabbit LR11 protein as a standard.

*Power calculations*. Group sizes were calculated using STPLAN software created by Barry Brown from the University of Texas MD Anderson Cancer Center. We have used mean values and standard deviations obtained from preliminary data from studies in *Lr11*^*−/−*^ mice. In this instance we expected effect sizes ranging down to 30% and aimed to obtain statistical significance of *P*<0.05 at a power of 0.9. With the expected s.d. of up to 12% and thus planned to include a minimum of seven animals/group for *in vivo* studies.

*Exclusion criteria*. Animals were excluded if their phenotypic characteristic fell more than two s.d. from the mean of the group. In practice only two animals were excluded from two separate parts of this study.

*Randomization and blinding*. No formal randomization was applied, but for diet studies, animals of each genotype were grouped so as to match mean starting bodyweights of chow and HFD groups. The researchers were not blinded as to the genotype of the animals as this was not practical because of the weight divergence phenotype. All measurement of gene expression and serum biochemistry was conducted by a researcher who was blinded for the experimental groups' identities.

### Statistics

Statistical analysis was performed with SPSS statistics version 23 (IBM) or Stat Flex v. 5.0 (Artech). Data was tested for normality and a Levene test for equality of variance was also applied to all data sets before parametric tests were applied. *P* values were not applied to exploratory initial studies where only the null hypothesis was being tested, only where an *a priori* hypothesis had been defined and informed the study. Associations of sLR11 levels with risk factors were examined by Pearson correlation analysis for continuous variables and by unpaired *t*-test for categorical variables between groups (sex, smoking, medication of diabetes, hypertension or dyslipidemia and history of coronary artery disease or cerebrovascular disease). The study subjects were principally separated to two groups based on the averaged values. In clinical studies, multiple linear regression analyses were used to calculate coefficient beta for sLR11 by controlling for age, smoking, HDL-cholesterol, HbA1c, BMI, VFA and SFA for study 1, and VFA and SFA for the study of patients that received bariatric surgery (study 3), which are significantly correlated with sLR11.

## Additional information

**Accession codes:** Microarray data have been deposited in the NCBI Gene Expression Omnibus (GEO) under accession codes GSE69117.

**How to cite this article:** Whittle, A. J. *et al*. Soluble LR11/SorLA represses thermogenesis in adipose tissue and correlates with BMI in humans. *Nat. Commun.* 6:8951 doi: 10.1038/ncomms9951 (2015).

## Supplementary Material

Supplementary Information Supplementary Figures 1-2 and Supplementary Tables 1-5.

## Figures and Tables

**Table 1:** Tissue weights from wild-type and *Lr11*^*−/−*^ mice.

	**Body (g)**	**Reproductive fat pad (g)**	**Inguinal fat pad (g)**	**Retroperitoneal fat pad (g)**	**Heart (g)**	**Liver (g)**	**Kidney (g)**
Tissue weights from group housed mice fed standard chow diet (age=8 weeks, *n*=10)							
WT	23.8±1.9	0.37±0.05	0.29±0.03	0.12±0.01	0.13±0.01	1.14±0.19	0.29±0.03
Lr11^−/−^	21.56±1.34	0.24±0.02	0.27±0.04	0.05±0.01	0.13±0.01	1.23±0.07	0.31±0.06
% of wild type	91	64	94	38	100	108	106
							
Tissue weights from group housed mice fed high-fat (60%) diet from 8-weeks-old mice (age=12 weeks, *n*=10)							
WT	34.13±4.53	2.19±0.45	1.77±0.26	0.81±0.15	0.13±0.01	1.43±0.17	0.33±0.02
LR11^−/−^	23.63±4.21	0.95±0.46	0.84±0.36	0.24±0.12	0.13±0.01	0.92±0.25	0.32±0.01
% of WT	69	43	47	30	100	64	96

WT, wild type.

**Table 2:** Correlation between sLr11 levels and characteristics of sleep apnoea.

**Single correlation with sLR11 levels**			**Multivariate analysis of correlates**	
**Factors**	***r*** **or mean±s.d.**	***P***	***β***	***P***
Age	0.169	**0.035**	0.034	0.084
Total cholesterol	0.039	0.626		
Triglyceride	0.062	0.443		
HDL-cholesterol	−0.196	**0.014**	−0.008	0.541
LDL-cholesterol	0.108	0.18		
BMI	0.186	**0.02**	0.047	0.548
VFA	0.212	**0.008**	0.008	0.056
SFA	0.206	**0.01**	0.001	0.83
HbA1c	0.171	**0.032**	0.080	0.736
CAD, yes/no	9.72±2.91/10.43±2.98	0.175		
CVD, yes/no	10.00±2.19/10.19±3.01	0.962		
Sex, men/women	10.08±2.91/10.39±3.10	0.489		
Smoking, yes/no	9.66±2.83/10.79±3.02	**0.025**	−0.482	0.19

BMI, body mass index; CAD, coronary artery disease; CVD, cerebrovascular disease; HDL, high-density lipoprotein; LDL, low-density lipoprotein; SFA, subcutaneous fat areas; VFA, visceral fat areas.

Initial correlations derived using Pearson correlation test. Multiple regression analysis performed in patients to examine the seven characteristics more strongly correlated with sLR11 levels using beta weights to detect significant differences in the contribution of each factor, *n*=156.

**Table 3:** Effect of bariatric surgery on adiposity and sLR11.

**Characterization pre- and 12 month post-bariatric surgery (*****n*****=14)**				
**Factor**	**Before**	**After**	***P***	**% reduction**
sLR11	16.79±7.03 ng ml^−1^	10.36±4.84 ng ml^−1^	**0.001**	37.6±11.9
BMI	44.37±8.64	31.43±5.41	**0.001**	28.3±9.7
VFA	245.30±135.44 cm^2^	96.84±57.44 cm^2^	**0.001**	57.7±20.9
SFA	651.23±439.63 cm^2^	356.50±129.76 cm^2^	**0.001**	37.1±20.5

**Correlation with % reduction in sLR11**				**Multiple linear regression**		
		**95% CI**			**95% CI**	
	***r***	**Lower**	**Upper**	***B***	**Lower**	**Upper**
% reduction of BMI	0.422	−0.140	0.778			
% reduction of VFA	0.583	0.076	0.851	0.211	−0.236	0.659
% reduction of SFA	0.561	0.043	0.841	0.165	−0.292	0.622

BMI, body mass index; CI, confidence interval; SFA, subcutaneous fat areas; VFA, visceral fat areas.

Changes to sLR11, BMI, VFA and SFA pre- and 12 months post-bariatric surgery with respective reductions (%). Data presented as means±s.d.; *P* values for each pre- and post-surgery comparison determined using a paired sample Wilcoxon Signed-Rank test, *n*=14. Contributions of factors to correlations with % reduction in sLR11 were assessed using multiple linear regressions.
