# Supplementary material for: Soluble LR11/SorLA represses thermogenesis in adipose tissue and correlates with BMI in humans
Source: Nat Commun. 2015 Nov 20;6:8951. doi: 10.1038/ncomms9951 (PMC4673879; doi:10.1038/ncomms9951)
Supplement: Supplementary Information — Supplementary Figures 1-2 and Supplementary Tables 1-5. [file ncomms9951-s1.pdf]

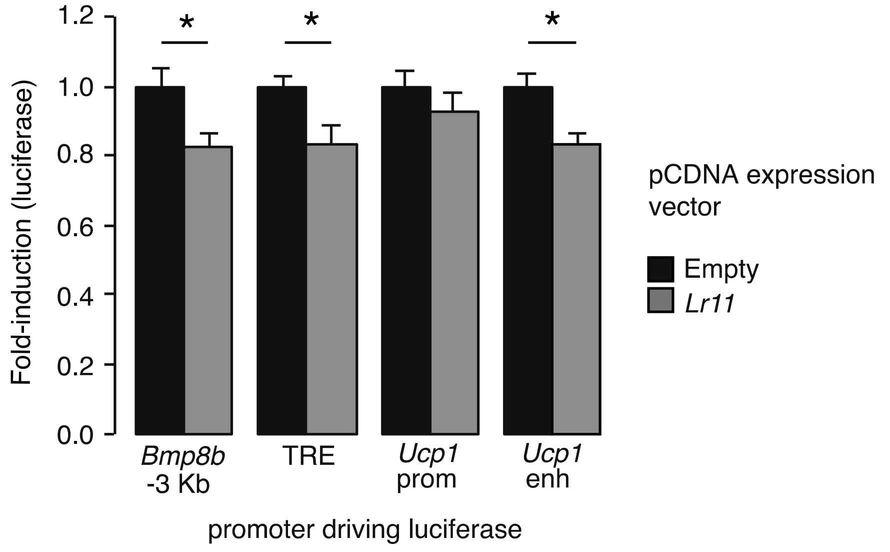

Supplementary Figure 1. Effect of LR11 overexpression on thermogenic gene promoters. Relative luciferase activity in HEK 293 cells transfected with a reporter driven by the indicated thermogenic gene promoter, enhancer or response element and co-transfected with an empty construct or one expressing LR11, TRE = Thyroid response element, BRE = BMP response element (n = 4) data presented as mean ± SEM, * = $p < 0.05$ obtained using one-way ANOVA followed by Tukey post-hoc test.

Fig. 5d

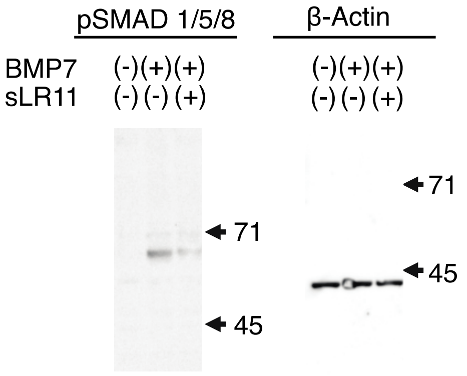

Fig. 5g

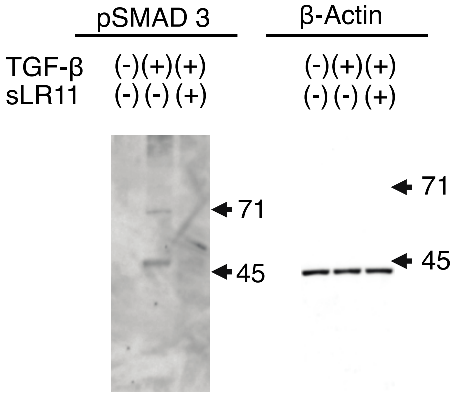

Fig. 5h

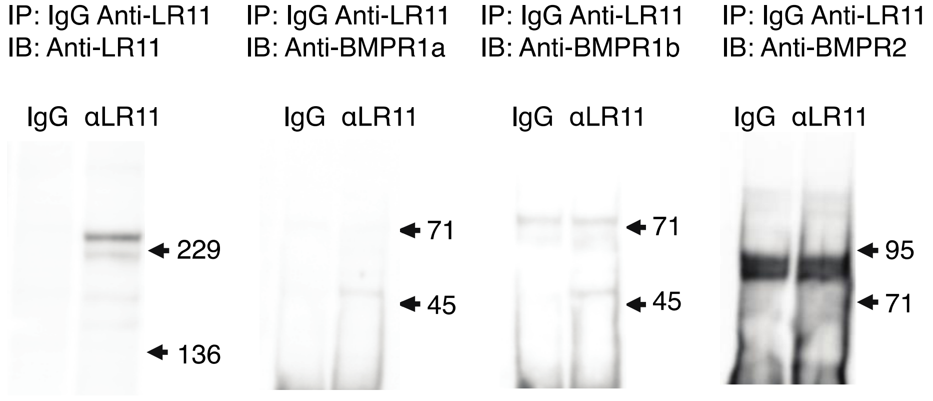

Supplementary Figure 2.Uncropped scans of blots. Western blots of indicated peptides and β-Actin as a loading control where relative quantities were compared (Fig. 5d & g). Weight markers are indicated in kDA and were acquired using Protein Multicolor III (Biodynamics) added to each sample well.

ID	Body Weight (g)	Inguinal fat (g)	Reproductive fat (g)	Liver (g)
WT1 chow	26.21	0.36	0.48	1.25
WT2 chow	25.15	0.32	0.38	1.12
WT3 chow	24.30	0.31	0.36	1.03
**Average**	**25.22**	**0.33**	**0.41**	**1.13**
SD	0.96	0.03	0.07	0.11
KO1 chow	23.69	0.34	0.31	1.16
KO2 chow	23.09	0.33	0.30	1.21
KO3 chow	22.23	0.31	0.29	1.15
**Average**	**23.00**	**0.33**	**0.30**	**1.18**
SD	0.73	0.02	0.01	0.03
WT1 HFD	33.10	1.45	1.54	1.36
WT2 HFD	29.21	1.01	1.28	1.09
WT3 HFD	28.73	0.91	0.99	1.22
**Average**	**30.35**	**1.12**	**1.27**	**1.22**
SD	2.40	0.29	0.27	0.14
KO1 HFD	24.30	0.52	0.56	1.03
KO2 HFD	25.20	0.75	0.89	1.02
KO3 HFD	22.96	0.49	0.55	0.86
**Average**	**24.15**	**0.59**	**0.67**	**0.97**
SD	1.13	0.14	0.19	0.10

Supplementary Table 1. Table of body and tissue weights of a supplemental group of 10 week old, male, single housed WT and LR11-/- mice.

Rank	gene symbol	log2 ratio	base gscale signals	Exp gscale signals	primary accession	probe ID
1	Cidea	15.04	33.1	1113426.6	NM_007702	A_51_P199168
2	Ucp1	13.97	57.0	913979.1	NM_009463	A_51_P426353
3	Cox8b	12.37	148.1	785178.5	NM_007751	A_52_P423814
4	Elovl3	11.19	15.5	36198.5	NM_007703	A_51_P324633
5	Thrsp	10.98	223.8	453599.6	NM_009381	A_55_P2018666
6	Acsm3	10.75	12.5	21559.0	NM_212441	A_51_P487175
7	Ppara	9.79	26.3	23312.9	NM_011144	A_55_P2071596
8	Slc27a2	9.55	24.4	18319.9	NM_011978	A_55_P2077153
9	Fabp3	9.35	986.9	642340.4	NM_010174	A_55_P1974367
10	Mfsd2a	9.18	16.2	9412.7	NM_029662	A_51_P279437
11	Ntrk3	9.12	15.1	8399.6	NM_182809	A_55_P2006808
12	LOC630751	9.06	14.2	7558.4	XR_142430	A_55_P1997253
13	Cyp2b10	8.92	13.5	6576.0	NM_009999	A_55_P2044653
14	Cox7a1	8.85	1428.3	658553.1	NM_009944	A_51_P148612
15	Poln	8.83	13.1	5957.5	NM_181857	A_55_P2080530
16	Gys2	8.69	76.3	31518.4	NM_145572	A_55_P1992555
17	Fasn	8.60	120.5	46910.9	NM_007988	A_52_P100252
18	Slc2a5	8.53	16.3	6041.6	NM_019741	A_51_P514405
19	Cpt1b	8.39	175.9	59074.9	NM_009948	A_55_P1956882
20	Otop1	8.23	85.6	25771.9	NM_172709	A_55_P2078143
21		8.21	134.4	39855.3	AK134220	A_66_P112394
22	Adrb3	8.14	348.3	98130.9	NM_013462	A_55_P1992849
23	9530008L14Rik	7.97	14.2	3562.5	NM_001145875	A_55_P2055742
24	E330011O21Rik	7.92	15.0	3637.5	NR_045698	A_55_P2257381
25	Pnpla3	7.91	41.8	10034.8	NM_054088	A_55_P2025514
26	Pank1	7.89	24.6	5855.3	NM_023792	A_51_P263591
27	Mpzl2	7.86	16.0	3721.8	NM_007962	A_52_P322421
28	Pik3c2g	7.43	15.5	2664.1	NM_207683	A_55_P1999953
29	S100b	7.42	285.2	48883.2	NM_009115	A_51_P468260
30	AW011956	7.41	13.7	2338.6	BC051535	A_55_P2319035
31	2310042D19Rik	7.38	21.7	3602.0	NM_172417	A_51_P322473
32	Clic5	7.35	122.6	19985.6	NM_172621	A_51_P420547
33	Cpn2	7.29	16.7	2612.2	NM_027904	A_51_P170562
34	Itih4	7.28	84.9	13213.4	NM_018746	A_55_P2077055
35	Lgr6	7.26	13.9	2132.1	AK040883	A_52_P75777
36	Tinag	7.24	16.3	2462.0	NM_012033	A_51_P283004
37	Plin5	7.24	715.6	108256.6	NM_001077348	A_55_P2104259
38		7.17	14.8	2125.4	ENSMUST00000060147	A_55_P1998535
39	Fbp2	7.10	14.3	1962.1	NM_007994	A_51_P499020
40	Ppargc1a	7.09	175.8	23899.2	NM_008904	A_51_P279038
41	Tpd52l1	7.07	12.7	1706.6	NM_009413	A_51_P482503
42	Apoc3	7.06	42.9	5736.1	NM_023114	A_55_P2117155

43	Acox2	6.96	22.0	2739.2	NM_053115	A_66_P138319
44	Ggnbp1	6.93	79.9	9758.6	ENSMUST00000142141	A_51_P158638
45		6.91	16.3	1954.1	ENSMUST00000064591	A_52_P478444
46	Slc2a4	6.89	915.1	108664.2	NM_009204	A_51_P217498
47	Gpd1	6.87	8282.5	968981.4	NM_010271	A_52_P16419
48	Ddo	6.85	54.3	6270.6	NM_027442	A_66_P123155
49	Acaca	6.84	15.1	1739.3	NM_133360	A_55_P2084703
50	Ccdc3	6.83	43.8	4972.2	NM_028804	A_51_P277431
51	Igsf21	6.82	72.5	8193.7	NM_198610	A_52_P305230
52	Acacb	6.80	52.3	5841.0	NM_133904	A_55_P2104532
53	Paqr9	6.74	61.9	6638.3	NM_198414	A_52_P450934
54	Pm20d1	6.72	12.7	1339.1	NM_178079	A_55_P2159585
55	Nrg4	6.71	181.1	18956.2	NM_032002	A_55_P2185905
56	C730029A08Rik	6.68	12.7	1306.8	AK083183	A_55_P2298319
57	Agbl4	6.68	23.8	2428.2	NM_030231	A_55_P2013569
58	Mal	6.62	32.2	3171.1	NM_010762	A_55_P2113160
59	Tuba8	6.57	150.3	14308.8	NM_017379	A_66_P119518
60	Nat8l	6.55	96.1	8980.5	NM_001001985	A_55_P1997821
61	Kcnk3	6.53	169.7	15714.1	NM_010608	A_52_P639402
62	Vwa3a	6.52	22.6	2082.2	NM_177697	A_66_P139647
63	Ces1d	6.49	1165.6	104979.2	NM_053200	A_51_P375969
64		6.44	53.4	4621.5	ENSMUST00000032900	A_51_P383755
65	Slc25a34	6.36	194.0	15913.5	NM_001013780	A_55_P2073248
66	A530016L24Rik	6.34	34.2	2761.9	NM_177039	A_55_P2138796
67	Chpt1	6.30	1718.3	135315.4	NM_144807	A_52_P655803
68	1110059M19Rik	6.26	26.8	2055.2	NM_026841	A_51_P477481
69	Adra1a	6.23	14.1	1060.6	NM_013461	A_52_P424778
70	Ehhadh	6.23	301.2	22649.6	NM_023737	A_51_P462918
71	Gpx5	6.17	16.6	1199.2	NM_010343	A_55_P2166758
72	Myo5b	6.17	13.8	991.5	NM_201600	A_52_P66130
73	Vldlr	6.11	106.3	7326.3	NM_013703	A_55_P2174836
74	2010003K11Rik	6.10	110.4	7557.7	NM_027237	A_51_P450278
75	Fn3k	6.08	30.4	2051.4	NM_022014	A_55_P2005859
76	Atp1a2	6.06	282.0	18802.0	NM_178405	A_55_P2030373
77	Trim67	6.05	15.9	1056.0	NM_198632	A_66_P114528
78	Cxadr	6.04	60.3	3968.8	NM_001025192	A_55_P1964638
79	Aldh3b2	6.04	31.5	2065.4	NM_001177438	A_55_P2006148
80	Grtp1	6.02	73.3	4755.9	NM_025768	A_51_P109144
81	Aspg	6.00	372.1	23896.4	NM_001081169	A_52_P338956
82	Esrrg	6.00	13.7	879.6	NM_001243792	A_65_P19089
83	Immp2l	5.99	134.7	8588.3	NM_053122	A_52_P517063
84	Rxrg	5.97	239.0	14966.5	NM_009107	A_51_P513311
85	Spag11b	5.96	13.4	834.3	NM_001034905	A_55_P2171768
86	Acot2	5.94	241.7	14819.2	NM_134188	A_52_P525183

87	Slc25a42	5.93	1149.2	70209.8	NM_001007570	A_55_P1955931
88	Tppp	5.92	15.5	939.2	NM_182839	A_51_P398260
89	Agpat2	5.92	1884.0	114202.5	NM_026212	A_55_P2125947
90	Decr1	5.90	261.7	15622.8	NM_026172	A_51_P208555
91	Txlnb	5.90	38.0	2267.0	NM_138628	A_51_P315391
92	Gm5144	5.89	177.1	10525.4	XR_105665	A_66_P118799
93	Serpina1a	5.86	98.9	5737.3	NM_001252569	A_55_P2165414
94	Ppp1r3c	5.86	205.5	11896.5	NM_016854	A_52_P30451
95	Bmp8b	5.84	15.2	869.5	NM_007559	A_55_P2012101
96	Dnajc22	5.83	14.8	845.6	NM_176835	A_51_P257743
97	Srl	5.83	16.6	942.6	NM_175347	A_55_P2126391
98	Acsl1	5.80	941.6	52306.0	NM_007981	A_51_P463452
99	Mlxipl	5.78	797.5	43795.6	NM_021455	A_55_P2119957
100	Gucy1b2	5.76	97.4	5284.7	NM_172810	A_51_P334281

Supplementary Table 2. The top 100 most regulated genes in Lr11 -/- ScWAT vs WT ScWAT, ranked in order of fold-change. Data taken from microarray, cluster 6. GEO accession number GSE69117. (See supplementary methods for further details).

gene	Forward	Reverse	probe
Beta3AR	CCAGCCAGCCCTGTTGA	GGACGCGCACCTTCATAGC	SYBR
Bmp8b	TCCACCAACCACGCCACTAT	CAGTAGGCACACAGCACACCT	CAGGCCCTGGTACATCTGATGAAGCC
Bmpr1alpha	Assay ID: Mm00477650_m1		
Bmpr1beta	Assay ID: Mm00432117_m1		
Cidea	GTGGACACAGAGGAGTTCTTT	GTCGAAGGTGACTCTGGCTATTC	ACAGAAATGGACACCGGG
Dio2	TGCGCTGTGTCTGGAACAG	CTGGAATTGGGAGCATCTTCA	SYBR
Elovl3	AAGGTTGTTGAACTGGGACGAC	GTGGTGGTACCAGTGGACAAA	SYBR
Hsl	GGAGCACTACAAACGCAACGA	TCGGCCACCGGTAAAGAG	CAGGCCTCAGTGTGACCGCCAGTT
Lr11	GTCTGCCCAATTACTTCCAC	TGACACTCGAACTCAAATCG	SYBR
Lrp1	Assay ID: Mm00464608_m1		
Pgc1alpha	AACCACACCCACAGGATCAGA	CTCTTCGCTTTATTGCTCCATGA	CAAACCCTGCCATTGTTAAGACCGAGAA
Ppara	GATTCAGAAGAAGAACCGGAACA	GCGAATTGCATTGTGTGACAT	TGCCGTTTTCACAAGTGCCTGTCTGTC
Prdm16	CAG CAC GGT GAA GCC ATT	GCG TGC ATC CGC TTG TG	SYBR
Smad7	Assay ID: Mm00484742_m1		
Sort1	Assay ID: Mm00490905_m1		
Ucp1	CCCGCTGGACACTGCC	ACCTAATGGTACTGGAAGCCTGG	AAGTCCGCCTTCAGATCCAAGGTGAAG
**Housekeepers**			
B2M	GGTCTTTCTGGTGCTTGTC	GTATGTTCGGCTTCCCATTC	SYBR
36B4	AGATGCAGCAGATCCGCAT	GTTCTTGCCCATCAGCACC	SYBR
18S	CGGCTACCACATCCAAGGAA	GCTGGAATTACCGCGGCT	GAGGGCAAGTCTGGTGCCAG
BetaAct	GCTCTGGCTCCTAGCACCAT	GCCACCGATCCACACAGAGT	ATCAAGATCATTGCTCCTCCTGAGCGC

Supplementary Table 3. Primer and probe sequences used for qRT-PCR. Assay numbers are provided for ABI assays on demand and where no probe was used, SYBR denotes SYBR green reagent used with primer sequences spanning intron boundaries.

Human Study 1 (sleep apnoea) Baseline Characteristics	
N	156
Age (yr)	62.9±9.5
Sex, men/women	110/46
BMI (kg/m^2)	24.9±3.8
Smoking, yes/no	85/71
Total cholesterol (mg/dl)	192.6±30.8
Triglyceride(mg/dl)	134.9±70.5
HDL-cholesterol (mg/dl)	53.1±14.3
LDL-cholesterol (mg/dl)	112.8±27.1
Visceral fat area (VFA) (cm2)	129.6±53.6
Subcutaneus fat area (SFA) (cm2)	156.4±89.2
HbA1c (%)	5.7±0.8
Diabetes medication (%)	16.7
Hypertension treatment (%)	59.0
Dyslipidemia medication (%)	35.9
Coronary artery diseases (CAD) (%)	35.9
Cerebrovascular diseases (CVD) (%)	6.4

Supplementary Table 4. Study 1 subjects – Sleep Apnoea. Patients are from Toho University Sakura Medical Center Clinic for sleep apnoea syndrome. Data presented as mean ± s.d. or proportion of subjects (%). Patients with sLR11 values >20 ng/mL were excluded due to the high possibility of accompanying malignant diseases and thus not included in the total n of this study group, n = 156.

Human Study 2 (Impared glucose tolerance)		correlation with sLR11 levels	
		**r**	**p**
N	25		
Age (yr)	47.28±7.43	-0.024	0.910
Male , men/women	18/7	0.136	0.517
BMI (kg/m^2)	33.27±10.76	0.640	**0.001**
Fasting plasma glucose (FPG) (mg/dl)	161.64±53.53	-0.150	0.473
Total cholesterol (mg/dl)	201.91±37.11	-0.122	0.563
Triglyceride (mg/dl)	223.74±102.94	-0.137	0.514
HDL-cholesterol (mg/dl)	42.74±9.92	-0.079	0.708
LDL-cholesterol (mg/dl)	114.02±27.61	0.065	0.756
HbA1c (%)	7.93±1.86	0.085	0.686
Systolic blood pressure (SBP) (mmHg)	130.6±14.40	-0.088	0.677
Diastolic blood pressure (DBP) (mmHg)	83.68±9.11	0.266	0.199

Supplementary Table 5. Study 2 subjects – impaired glucose tolerance. Subjects are patients in Toho University Sakura Medical Center Clinic for Diabetes and Obesity or in Yanbian University Hospital Department of Diabetes. Patients do not suffer from malignant diseases or Alzheimer's disease. Data presented as mean ± s.d. Correlation assessed using Spearman rank correlation.
